# *WATER-SOAKED SPOT1* Controls Chloroplast Development and Leaf Senescence *via* Regulating Reactive Oxygen Species Homeostasis in Rice

**DOI:** 10.3389/fpls.2022.918673

**Published:** 2022-05-26

**Authors:** Jiangmin Xu, Zhiyuan Ji, Chunlian Wang, Feifei Xu, Fujun Wang, Yuhan Zheng, Yongchao Tang, Zheng Wei, Tianyong Zhao, Kaijun Zhao

**Affiliations:** ^1^National Key Facility for Crop Gene Resources and Genetic Improvement, Institute of Crop Sciences, Chinese Academy of Agricultural Sciences, Beijing, China; ^2^State Key Laboratory of Crop Stress Biology for Arid Areas, College of Life Sciences, Northwest A&F University, Xianyang, China; ^3^Rice Research Institute, Guangdong Academy of Agricultural Sciences, Guangzhou, China

**Keywords:** transmembrane kinases, water-soaked spot, leaf senescence, chloroplast development, reactive oxygen species, rice

## Abstract

Transmembrane kinases (TMKs) play important roles in plant growth and signaling cascades of phytohormones. However, its function in the regulation of early leaf senescence (ELS) of plants remains unknown. Here, we report the molecular cloning and functional characterization of the *WATER-SOAKED SPOT1* gene which encodes a protein belongs to the TMK family and controls chloroplast development and leaf senescence in rice (*Oryza sativa* L.). The *water-soaked spot1* (*oswss1*) mutant displays water-soaked spots which subsequently developed into necrotic symptoms at the tillering stage. Moreover, *oswss1* exhibits slightly rolled leaves with irregular epidermal cells, decreased chlorophyll contents, and defective stomata and chloroplasts as compared with the wild type. Map-based cloning revealed that *OsWSS1* encodes transmembrane kinase TMK1. Genetic complementary experiments verified that a Leu396Pro amino acid substitution, residing in the highly conserved region of leucine-rich repeat (LRR) domain, was responsible for the phenotypes of *oswss1*. *OsWSS1* was constitutively expressed in all tissues and its encoded protein is localized to the plasma membrane. Mutation of *OsWSS1* led to hyper-accumulation of reactive oxygen species (ROS), more severe DNA fragmentation, and cell death than that of the wild-type control. In addition, we found that the expression of senescence-associated genes (SAGs) was significantly higher, while the expression of genes associated with chloroplast development and photosynthesis was significantly downregulated in *oswss1* as compared with the wild type. Taken together, our results demonstrated that OsWSS1, a member of TMKs, plays a vital role in the regulation of ROS homeostasis, chloroplast development, and leaf senescence in rice.

## Introduction

Plant leaf is photosynthetic organ for energy production and nutrient assimilation during plant growth. Plant leaf senescence is the final stage of leaf development and controlled by programmed cell death (PCD) (Lim et al., 2007). During leaf senescence, reactive oxygen species (ROS) accumulate, malondialdehyde (MDA) content increases, the antioxidant enzyme activity decreases, chlorophyll degrades, photosynthesis efficiency reduces, and cell membrane gets damaged (Lim et al., 2007; Woo et al., 2019; Domínguez and Cejudo, 2021). ROS, including singlet oxygen (^1^O_2_), superoxide anions (O_2_^–^), hydroxyl radicals (OH), and hydrogen peroxide (H_2_O_2_), are important signaling molecules that trigger PCD and leaf senescence in plants (Wang et al., 2013; Mittler, 2017). Hyper-accumulation of ROS can disrupt the redox balance in cells and cause severe damage to lipids, proteins, and DNA. In addition, ROS affect the fluidity of biofilm, resulting in enzyme inactivation (Foyer and Noctor, 2005; Saed-Moucheshi et al., 2014). Therefore, production and scavenging of ROS must be tightly controlled by the antioxidant defense system to maintain a dynamic balance of the ROS level in plants (Lee S. et al., 2012; Mittler, 2017). The major antioxidant enzymes are catalase (CAT), superoxide dismutase (SOD), peroxidase (POD), and ascorbate peroxidase (APX) (Saed-Moucheshi et al., 2014).

In agriculture, plant leaves undergo normal leaf senescence positively contributes to crop yield by transferring its photosynthetic products into grains or other crop harvest organs (Wu et al., 2012). Early leaf senescence (ELS) means premature leaf yellowing and/or withering compared to wild type during reproductive growth, which decreases the functional period of leaves, thus affecting crop yield and quality (Lim et al., 2007). Leaf senescence is associated with the expression of specific genes (Woo et al., 2019). More than 185 senescence-associated genes (SAGs) have been identified in rice (Li et al., 2020). These genes are mainly associated with chloroplast development (Yang Y. et al., 2016; Chen et al., 2021), chlorophyll degradation (Kusaba et al., 2007; Morita et al., 2009; Jiang et al., 2011; Yamatani et al., 2013; Cui et al., 2021), hormone signal transduction (Kong et al., 2006; Chen et al., 2013; Liang et al., 2014), protease transport metabolism (Wu et al., 2016; Hong et al., 2018), and energy transport metabolism (Huang et al., 2007; Qiao et al., 2010).

*Receptor-like kinases* (*RLKs*) consist in one of the ubiquitous and most abundant gene families in plants. There are more than 1130 *RLK* genes in rice, twice as many as in *Arabidopsis* due to gene duplication (Shiu et al., 2004). A typical RLK is composed of a variable extracellular domain to perceive specific ligands, a transmembrane domain to connect the extracellular and intracellular parts for signal transmission, and an intracellular domain to transduce external signals into the cell to activate or inactivate the downstream regulatory components *via* phosphorylation (Morillo and Tax, 2006; Gish and Clark, 2011). The common structural element of many plant RLKs is an extracellular leucine-rich repeats (LRRs) domain, often referred to as LRR-RLK (Kinoshita et al., 2005). Each LRR is typically 20–30 residues long that has a high portion of leucine and other hydrophobic residues. The number of LRR motifs ranges between 3 and 27, and the distribution interval of LRR varies in different LRR-RLKs (Bella et al., 2008).

The transmembrane kinases (TMKs) of LRR-RLKs family play an important role in regulating plant growth and development (Xu et al., 2014). In *Arabidopsis*, unique double and triple TMK mutant combinations lead to serious growth defects, such as late flowering and seed sterility (Dai et al., 2013). The *Arabidopsis* TMK4 acts as a node in auxin and ABA signaling pathways (Wang et al., 2020; Li et al., 2021). In addition, TMKs have vital roles in non-canonical auxin signaling in regulating pavement cell morphogenesis, differential growth of the apical hook, lateral root formation, root gravitropic response, and thermomorphogenesis in *Arabidopsis* (Lin et al., 2021). Up to the present, only one rice TMK-encoding gene (*OsTMK*) has been characterized, and it is involved in gibberellin signal transduction (van der Knaap et al., 1999). Whether rice TMKs play a role in regulation of leaf senescence remains unknown.

In this study, we isolated and characterized the rice mutant *water-soaked spot1* (*oswss1*), which exhibits water-soaked spots and necrotic symptoms on leaves. *OsWSS1* encodes a member of the TMK subfamily of RLKs. The single amino acid substitution (Leu396Pro) in OsWSS1 results in the water-soaked spots and early leaf senescence phenotypes of the mutant *oswss1*. These results demonstrated that *OsWSS1* encodes a TMK that plays an important role in regulation of rice leaf senescence.

## Materials and Methods

### Plant Materials and Growth Conditions

The rice early leaf senescence mutant *oswss1* was identified from the ethyl methane sulphonate (EMS) mutagenesis library of the *indica* rice cultivar JG30. The mutant *oswss1* was crossed with two *japonica* cultivars (02428 and Nipponbare) and the *indica* line JG30 for genetic analysis and gene mapping. The F_1_ plants were self-pollinated to produce F_2_ seeds. Unless specified otherwise, all parents, F_1_ hybrids, F_2_ populations, and transgenic plants were grown under natural conditions in paddy fields in Beijing and Hainan province, China.

### Measurement of Chlorophyll Concentrations and Relative Water Content

For determination of the content of chlorophyll *a* (Chl *a*), chlorophyll *b* (Chl *b*), and carotenoid (Car), 10–30 mg wild-type leaves and *oswss1* green leaves and water-soaked spot leaves were cut into small pieces and chlorophylls were extracted in 95% ethanol solution for 48 h at 4°C in the dark. The absorbance values at wavelengths of 665, 649, and 470 nm were measured using a BioPhotometer plus (Eppendorf, Germany). The 95% ethanol was used as a blank control. The concentrations of Chl *a*, Chl *b*, and Car were calculated following a published method (Lichtenthaler, 1987). There are six biological replicates for each group.

For measurement of RWC, the fully expanded leaves of wild type and *oswss1* were excised from plants in the field, and the fresh weight was measured and recorded as m1. The leaves were soaked in distilled water in a 50 mL centrifuge tube for 2 h, weighed, and recorded as m2. The leaves were then dried in a 60°C drying oven for 48 h, weighed, and recorded as m3. The percentage of RWC was calculated as (m1 − m3)/(m2 − m3) × 100.

### Transmission Electron Microscopy and Scanning Electron Microscopy

Wild-type leaves, *oswss1* green leaves, and water-soaked spot leaves were cut into pieces and were immersed in 2.5% pre-cooled glutaraldehyde solution, vacuumed for 60 min, and then kept at 4°C overnight. Subsequent procedures were performed following a published protocol (Zheng et al., 2021). Briefly, the samples were washed with phosphate buffer (0.1 M, pH 7.0) and fixed with 1% osmic acid stationary solution (pH 7.3), then dehydrated using a graded ethanol series. After dehydration, samples were embedded in epoxy resin. Afterward, sections were stained with uranyl acetate and examined with a Hitachi HT7700 transmission electron microscope.

For scanning electron microscopy analysis of the leaf epidermis, the samples were fixed in 2.5% glutaraldehyde solution and washed using phosphate buffer, and then dehydrated through a graded ethanol series. The samples were dried by the critical-point drying method and coated with gold, and then observed using a Hitachi TM-1000 scanning electron microscope (Rao et al., 2015).

### Observation of Leaf Cell Morphology

At the tillering stage (87 days after sowing, DAS), leaves of wild type and *oswss1* mutant were fixed with formalin/acetic acid/alcohol fixative (FAA, 3.7% formaldehyde, 5% glacial acetic acid, and 50% ethanol) for 24 h, then dehydrated with 40, 60, 80, and 100% alcohol for 50 min, respectively. After that, the leaves were placed in 1.25 g/mL chloral hydrate solution for 60 min at 96°C. The upper epidermal tissues of leaves were scraped, and the lower epidermal cells were examined and photographed using BX43 light microscope (Tokyo, Japan).

For paraffin sectioning, leaves of wild type and *oswss1* mutant were fixed in FAA fixative overnight at 4°C. The paraffin sectioning procedures were performed following a published protocol (Ren et al., 2016). In brief, the samples were dehydrated with graded ethanol series, infiltrated with graded xylene series, and embedded in paraffin. The embedded samples were sliced into 10 μm thickness pieces and stained with a solution containing 1% safranine and 1% Fast Green. The sections were observed and photographed using BX43 light microscope (Tokyo, Japan).

### Map-Based Cloning of *OsWSS1*

The phenotypes of F_1_ plants and the segregation ratio of F_2_ population were identified for genetic analysis. F_2_ individuals with mutant phenotype were selected for DNA extraction and gene mapping. The parents and 21 F_2_ individuals with mutant phenotype from the 02428/*oswss1* cross were subjected to initial linkage analysis by genotyping using 189 polymorphic insertion/deletion (InDel) markers covering 12 rice chromosomes. Subsequently, a large F_2_ population with 1184 mutant individuals were used for fine mapping. New polymorphic primers were developed based on DNA sequence differences between the *japonica* cultivar Nipponbare (NIP) and *indica* cultivar JG30 to further narrow down the *OsWSS1* locus. The wild type and *oswss1* mutants were amplified by PCR using the marker primers (Supplementary Table 2). The results were analyzed using DNAMAN software.

For complementary experiment of *oswss1*, a 10 kb genomic DNA fragment containing the 6.6 kb wild type *OsWSS1* coding sequence (CDS), 2.3 kb upstream and 1.1 kb downstream, was amplified by PCR using primers pWSS1-CF and pWSS1-CR (Supplementary Table 3). The amplified fragment was cloned into the binary vector pCAMBIA1300 using the ClonExpress^®^ II One Step Cloning Kit (Vazyme, China). The constructed vector was transformed into callus cells of *oswss1* by *Agrobacterium tumefaciens*-mediated transformation and the transgenic plants were regenerated.

### Subcellular Localization

To detect the subcellular localization of OsWSS1, the full-length CDS of *OsWSS1* without the termination codon was amplified by PCR using primers WSS1-GFP-F and WSS1-GFP-R (Supplementary Table 3) and fused into the N-terminal of enhanced green fluorescent protein (eGFP) in the pCAMBIA1300 vector under the control of the CaMV 35S promoter to generate a *p35S*:*OsWSS1*:*eGFP* vector. The constructs were then transiently expressed or co-expressed with PIP2-mCherry, a plasma membrane marker, in rice protoplasts derived from the wild-type rice seedlings following a published protocol (Rao et al., 2015). In addition, the constructs were transiently expressed or co-expressed in *Nicotiana benthamiana* leaves by *Agrobacterium tumefaciens*-mediated infiltration following the methods described previously (Gao et al., 2016). GFP fluorescence signals in rice protoplasts and epidermal cell of *N. benthamiana* leaves were observed using a Zeiss LSM700 laser scanning confocal microscope.

### RNA Extraction and Quantitative Real-Time PCR

Total RNA was extracted using the RNAiso Plus reagent (Takara, Japan) according to the manufacturer’s manual. Total of 1 μg RNA was reversely transcribed into complementary DNA (cDNA) using a FastQuant RT Kit with gDNA remover (Tiangen, China). Quantitative reverse transcription PCR (qRT-PCR) was performed on an ABI 7500 Real-Time PCR system (Life Technologies, Carlsbad, CA, United States) with an SYBR^®^ Premix ExTaq™ II (Takara). Rice *OsActin* (*LOC_Os03g50885*) was used as an internal reference gene. The relative quantitative analysis was performed by 2^–Δ^ ^Δ*CT*^ method (Livak and Schmittgen, 2001). Primers used for qRT-PCR are listed in Supplementary Table 4.

### Measurement of Hydrogen Peroxide (H_2_O_2_) and Malondialdehyde Contents, and Antioxidants Activities

At the tillering stage, fresh leaves (0.3 g) of wild type and *oswss1* were collected and ground into homogenate in a precooled mortar with 2 mL 50 mmol L^–1^ precooled sodium phosphate buffer (pH 7.8). The homogenate was centrifuged at 12, 000 × *g* at 4°C for 10 min. The supernatant was collected for determination of the content of H_2_O_2_ and MDA, the activities of SOD and CAT, using the kits (A064-1-1, A003-1-2, A001-1-2, and A007-1-1) following the manufacturer’s manuals (Nanjing Jiancheng Bioengineering Institute, China).

### Histochemical Analysis and TUNEL Assay

The 3,3′-diaminobenzidine (DAB) and nitroblue tetrazolium (NBT) staining assays were performed as described previously (Thordal-Christensen et al., 1997). Briefly, leaves of wild type and *oswss1* were collected at the tillering stage, then soaked in 0.1% (w/v) DAB (Sigma) or 0.05% (w/v) NBT (Duchefa) solution and dyed for more than 8 h under dark conditions. The staining leaves were transferred into 95% ethanol for decolorization. Trypan blue staining was used to detect dead cells according to the method of a published protocol (Zheng et al., 2021). In brief, leaves at the same part were cut off and immersed in trypan blue solution and boiled for 10 min. After being kept in dark overnight, the leaves were transferred to 25 mg/mL chloral hydrate to decolorize for 3 days.

Terminal deoxynucleotidyl transferase dUTP nick-end labeling (TUNEL) assay was performed following a published protocol (Huang et al., 2007). Briefly, leaves of wild type and *oswss1* were collected and fixed in FAA solution for 24 h, then embedded in paraffin and cut into thin slices. Slides were hydrated with ethanol series and treated with proteinase K in phosphate buffer (pH 7.4). The TUNEL assay was performed using a Fluorescein *In Situ* Cell Death Detection Kit (Roche) according to the manufacturer’s instructions.

### Bioinformatics Analyses

OsWSS1 protein transmembrane domain prediction was performed using TMHMM server v. 2.0^1^. Conserved domains of the OsWSS1-encoded proteins were analyzed using SMART^2^. The sequences used for phylogenetic analysis were obtained from the NCBI Blast website^3^ using OsWSS1 protein sequence (BAF28192) as the query. Alignment of the full-length amino acid sequences was performed using the DNAMAN software, and sequence logos of each protein were generated online with WebLogo3 (Crooks et al., 2004). The phylogenetic trees were constructed using MAGE 7.0 version software using the bootstrap method with 1000 bootstrap replicates. Protein sequence alignment was performed among OsWSS1 and its homologs from *Oryza brachyantha* (XP_006663422.1), *Sorghum bicolor* (XP_002450707.1), *Setaria italic* (XP_004979222.1), *Zea mays* (XP_008670729.1, PWZ25297.1), *Panicum hallii* (XP_025827113.1), *Triticum dicoccoides* (XP_037421068.1), *Triticum aestivum* (KAF7042763.1), *Aegilops tauschii* subsp. *tauschii* (XP_020188972.1), *Brachypodium distachyon* (XP_003576062.1), *Panicum miliaceum* (RLM70345.1), *Ananas comosus* (OAY69618.1), *Triticum urartu* (EMS63078.1), *Triticum turgidum* subsp. *durum* (VAH91395.1), *Hordeum vulgare* (KAE8766681.1), *Musa balbisiana* (THU51216.1), *Carex littledalei* (KAF3334352.1), *Phoenix dactylifera* (XP_008812470.1), *Capsicum annuum* (XP_016551399.1), *Cocos nucifera* (AID55112.1), *Cannabis sativa* (XP_030507417.1), *Vitis vinifera* (RVW82026.1), *Camellia sinensis* (XP_028113257.1), *Helianthus annuus* (XP_021981103.1), *Medicago truncatula* (XP_003601704.1), *Nelumbo nucifera* (XP_010246532.1), and *Arabidopsis thaliana* (AAP04161.1).

## Results

### The *Oswss1* Mutant Exhibits Water-Soaked Spots and Withered Leaves

**FIGURE 1:**
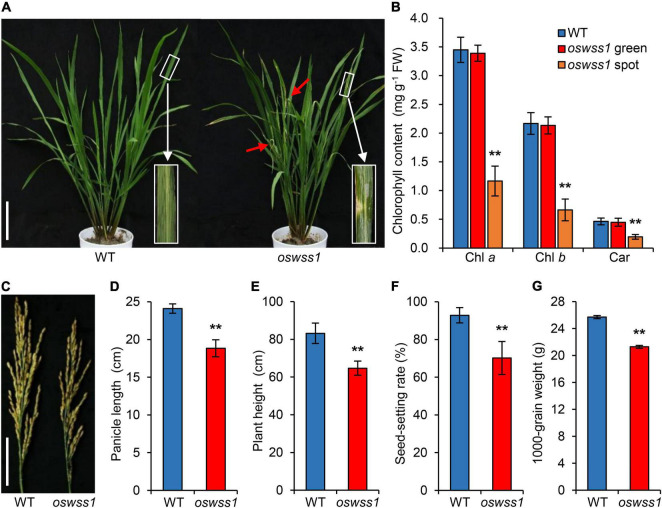
Phenotype characterization and major agronomical traits of the rice *oswss1* mutant. **(A)** Phenotype of wild type (WT) and *oswss1* at the initial tillering stage (60 days after sowing, DAS). Inset shows a magnified view of a water-soaked spot of *oswss1* leaves. Scale bar = 14 cm. **(B)** Comparison of chlorophyll content between WT and *oswss1* at the initial tillering stage (60 DAS). Values are mean ± SD (*n* = 6). **(C)** Panicle morphology comparison between WT and *oswss1* at the heading stage (118 DAS). Scale bar = 6 cm. **(D–G)** Agronomic traits of WT and *oswss1* plants. **(D)** Panicle length; **(E)** plant height; **(F)** seed-setting rate; **(G)** 1,000-grain weight. Values are mean ± SD (*n* = 10). **P < 0.01 (Student’s *t*-test).

The rice *oswss1* mutant was isolated from the *indica* rice cultivar JG30 that was treated with EMS mutagenesis. Under natural conditions of the paddy field, light green water-soaked spots gradually appeared on *oswss1* leaves at the initial tillering stage (60 days after sowing, DAS), the water-soaked spot leaves gradually withered, while the wild-type (WT) leaves remain green (Figure 1A and Supplementary Figures 1A–C). An abundance of light green spots appeared on the *oswss1* leaves at the booting stage (105 DAS, Supplementary Figure 1D). We measured the chlorophyll (Chl) content of the water-soaked spots and green parts of *oswss1* leaves at the initial tillering stage. The results showed no significant difference between the green part of *oswss1* and WT leaves, while the contents of Chl *a*, Chl *b*, and Car in the water-soaked zone of *oswss1* leaves were significantly lower than those in the WT leaves (Figure 1B). At the heading stage (118 DAS), the panicle length of *oswss1* was shorter than that of the WT (Figures 1C,D). The lengths of the internodes I, II, III, IV, and V of *oswss1* were shortened by 22.6, 30.2, 33.8, 22.5, and 32.8%, respectively, in comparison with WT (Supplementary Figure 2). In addition, the plant height, seed-setting rate, and 1000-grain weight were lower in *oswss1* than that of the WT (Figures 1E–G). In short, the water-soaked spots of *oswss1* have an obvious negative effect on the agronomic traits.

### Irregular Cell Structure and Abnormal Chloroplast Development in the *Oswss1* Leaves

**FIGURE 2:**
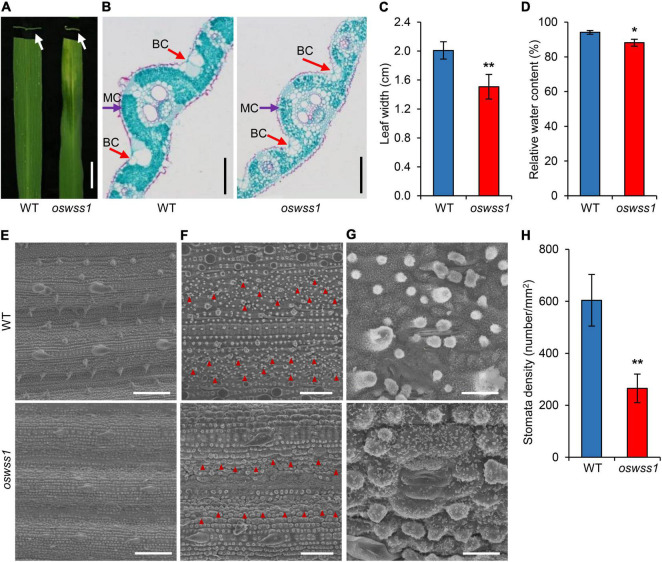
Morphology comparison between wild type (WT) and *oswss1* leaves at the tillering stage (87 DAS). **(A)** Close-up image and cross section of WT and *oswss1* mutant leaves. Arrows indicate the cross section of water-soaked spot leaf. Scale bar = 2 cm. **(B)** Comparison of cell structure between WT and *oswss1* leaves. BC, bulliform cell; MC, mesophyll cell. Scale bars = 200 μm. **(C)** Comparison of leaf width between WT and *oswss1* leaves. **(D)** Comparison of relative water content between WT and *oswss1* leaves. **(E)** Scanning electron microscopy analysis of leaf morphological characteristics between WT and *oswss1* leaves. Scale bars = 100 μm. **(F)** Comparison of stomata density between WT and *oswss1* leaves. The red triangles indicate the positions of stomata. Scale bars = 50 μm. **(G)** Morphological characteristics of stomata between WT and *oswss1* leaves. Scale bars = 10 μm. **(H)** Statistical analysis of stomata density between WT and *oswss1*. Values are mean ± SD (*n* = 6). *P < 0.05; **P < 0.01 (Student’s *t*-test).

At the maximum tillering stage (87 DAS), the water-soaked zone of *oswss1* leaves were slightly curled (Figure 2A). Paraffin sectioning assay showed that the mesophyll cells were arranged normally and regularly in the WT leaves (Figure 2B). In contrast, the volume of bulliform cells (BCs) in the water-soaked zone of *oswss1* leaves was significantly reduced; the mesophyll cells (MCs) were abnormal, and the cell arrangement was irregular (Figure 2B). The leaf width of the water-soaked zone of *oswss1* mutant was narrower than that of WT (Figure 2C). In addition, the relative water content (RWC) of the water-soaked zone of *oswss1* leaves was lower, compared with that of WT leaves (Figure 2D). These results implied that the slightly rolled *oswss1* leaves may be caused by the atrophied BCs and MCs.

Scanning electron microscopy (SEM) assays showed the leaf surface in the water-soaked zone of *oswss1* leaves was seriously shrunk, the cuticular papillae were reduced, the stomatal morphology was deformed, and the stomatal density was also significantly reduced compared with that of WT (Figures 2E–H). Furthermore, the lower epidermal cells of the leaves were observed under light microscope. The leaf epidermal cells of WT showed regular rectangular shape, tightly arranged and had a complete cell structure (Supplementary Figure 3A). However, the leaf epidermal cell shape in the water-soaked zone of *oswss1* leaves was irregular, the cell arrangement was loose, and the cell structure changed significantly (Supplementary Figure 3B). Overall, these results suggested that *OsWSS1* play a role in regulation of leaf development.

**FIGURE 3:**
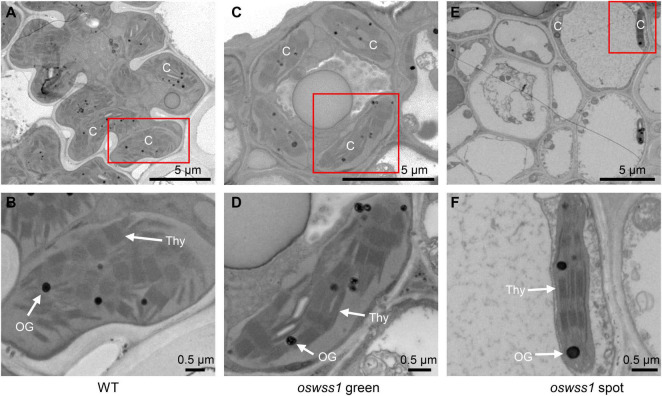
Comparison of chloroplast ultrastructural between wild type (WT) and *oswss1* leaves at the tillering stage (87 DAS). Transmission electron microscopy (TEM) was used to detect the chloroplasts ultrastructural in WT leaves **(A,B)**, *oswss1* green sectors **(C,D)** and *oswss1* spot sectors **(E,F)**. C, chloroplast; OG, osmiophilic plastoglobuli; Thy, thylakoid. The red box indicates the area magnified.

The chloroplast ultrastructure of the WT leaves, the green parts and water-soaked zone of *oswss1* leaves were compared by transmission electron microscopy (TEM) at the tillering stage (87 DAS). TEM observation revealed that the chloroplasts were normal and structural integrity in the WT and the green part of *oswss1*, including intact membrane and dense grana lamella (Figures 3A–D). In contrast, in the water-soaked zone of *oswss1* leaves, the chloroplast development was impaired, the thylakoid lamellar structure was abnormal, and the grana lamellar was severely reduced, the number of chloroplasts was decreased (Figures 3E,F). The results suggested that *OsWSS1* plays an important role in chloroplast development.

### Map-Based Cloning of *OsWSS1*

The *oswss1* was crossed with two *japonica* rice cultivars (NIP and 02428) and the *indica* line JG30, respectively. The leaves of F_1_ plants were the same as the WT. The phenotype of leaf morphology showed 3:1 (WT: mutant) segregation ratio in the F_2_ populations (Supplementary Table 1). These results indicated that the water-soaked spot phenotype of *oswss1* was controlled by a single recessive gene.

**FIGURE 4:**
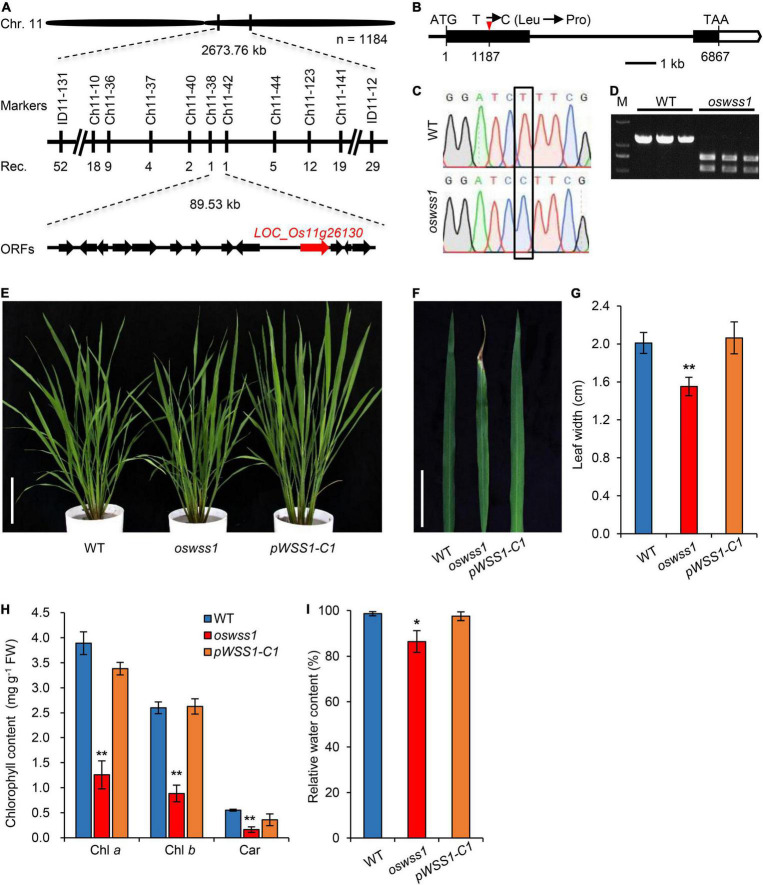
Map-based cloning of *OsWSS1* and complementation test. **(A)** Fine mapping of *OsWSS1*. The *OsWSS1* locus was mapped to an 89.53 kb genomic region on chromosome 11 between markers Ch11-38 and Ch11-42. The molecular markers and recombinant numbers are displayed. **(B)** The structure and mutation site in *OsWSS1*. The white boxes, black boxes, and black lines represent untranslated regions, exons, and introns, respectively. **(C)** DNA sequencing to confirm the point mutation of the *OsWSS1* in the *oswss1* mutant. The black box indicates the mutation position. **(D)** Confirmation of the mutation by cleaved amplified polymorphic sequence. PCR products from *oswss1* were digested by *Bam*HI while PCR products from wild type (WT) were not digested by *BamH*1. **(E)** The plant morphology among WT, *oswss1*, and complementation transgenic line at the initial tillering stage (60 DAS). Scale bar = 15 cm. **(F)** Leaf morphology among WT, *oswss1* and complementation transgenic line at the tillering stage. Scale bar = 5 cm. **(G)** Comparison of leaf width of WT, *oswss1*, and complementation plants. **(H)** Comparison of chlorophyll content of WT, *oswss1*, and complementation plants. **(I)** Relative water content among WT, *oswss1*, and complementation leaves. Values are mean ± SD (*n* = 6). *P < 0.05, **P < 0.01 (Student’s *t*-test).

For molecular cloning of *OsWSS1* locus, the polymorphism between the parents of *oswss1* and 02428 was analyzed, a total of 189 pairs of polymorphic insertion/deletion (InDel) markers were screened. Subsequently, 21 mutant individuals from the 02428/*oswss1* F_2_ populations were used for linkage analysis. The *OsWSS1* locus was preliminarily mapped on chromosome 11 between the markers ID11-131 and ID11-12 (Figure 4A). For fine mapping of *OsWSS1*, nine new InDel markers were developed (Supplementary Table 2). Through genotyping of 1184 mutant individuals from the F_2_ populations, *OsWSS1* was narrowed down to an 89.53 kb region between the markers Ch11-38 and Ch11-42 (Figure 4A). This region contains 13 annotated open reading frames (ORFs). Sequencing of these 13 ORFs revealed a T-to-C single-base substitution in the first exon of *LOC_Os11g26130*. This T to C change results in an amino acid substitution from leucine (Leu) to proline (Pro) in the 396th residue (Figures 4B,C). Additionally, the mutation of the *OsWSS1* gene was verified by a cleaved amplified polymorphic sequence (CAPS) marker which was developed based on this point mutation (Figure 4D).

The genetic complementation was performed to confirm that the mutation in *LOC_Os11g26130* is responsible for the *oswss1* mutant phenotype. The complementation vector pCAMBIA1300:*OsWSS1* with a WT genomic fragment containing the entire coding region of *OsWSS1* along with 2.3 kb upstream sequence and 1.1 kb downstream sequence was constructed and transformed into the *oswss1*-derived callus through *Agrobacterium tumefaciens*-mediated transformation. A total of 25 transgenic lines were generated, among them, 19 independent and positive complementary lines (including *pWSS1-C1*) exhibited the WT normal leaf phenotype (Figures 4E,F and Supplementary Figure 4A). Sequencing revealed that the *pWSS1-C1* transgenic line was heterozygous for the *oswss1* mutation in the T0 transgenic plant (Supplementary Figure 4B). There was no significant difference in the leaf width, chlorophyll content, and RWC between the WT and *pWSS1-C1* complementary lines (Figures 4G–I). Furthermore, the plant height, panicle length, 1,000-grain weight, and seed-setting rate of 10 *pWSS1-C* complementary lines tested were recovered to the WT level (Supplementary Figures 5A–E and Supplementary Table 5). Quantitative real-time PCR (qRT-PCR) analysis revealed that expression of *OsWSS1* in *oswss1* was similar to that of the WT plants, whereas the relative transcript level of *OsWSS1* was markedly increased in the *pWSS1-C1* compared with that in the WT and *oswss1* plants (Supplementary Figure 5F), indicating that the phenotype of *oswss1* is not caused by the difference in *OsWSS1* expression. Taken together, these data confirmed that the mutation of *LOC_Os11g26130* was responsible for the early leaf senescence phenotype of *oswss1*.

### *OsWSS1* Encodes a TMK1 Type LRR-RLK

**FIGURE 5:**
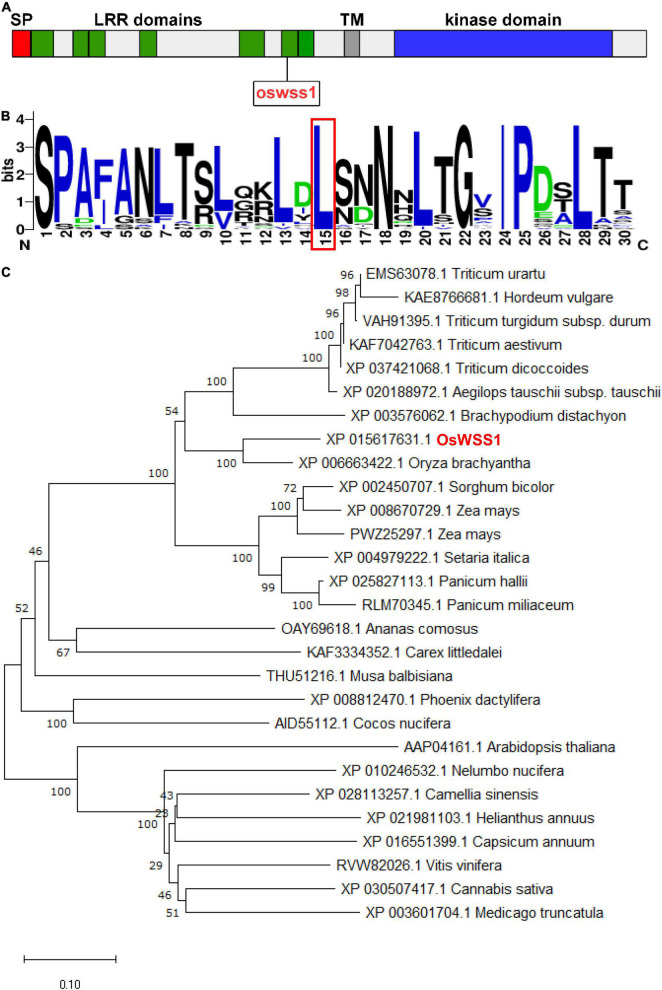
Phylogenic analysis of OsWSS1. **(A)** Schematic diagram of the OsWSS1 protein. SP, signal peptide; LRR domain, leucine-rich repeat region; TM, transmembrane domain. The rectangle indicates the position of the point mutation of the *OsWSS1* in the *oswss1* mutant. **(B)** Conservation analysis of the amino acid substitution region and the frequency of the 50 homologous genes. The red-boxed region indicates the position of the amino acid transition. **(C)** Phylogenetic tree of the OsWSS1 protein. OsWSS1 is highlighted in red. The phylogenetic tree was constructed using MEGA 7.0 with the neighbor-joining method and 1,000 bootstrap replicates.

According to the RAP-DB database^4^, the coding sequence of *OsWSS1* is composed of 2 exons with 2739 bp of cDNA sequence. Using the deduced OsWSS1 protein sequence as a query to search on the NCBI database^5^, an identical protein which annotated as receptor protein kinase TMK1 was found. TMK1 belongs the LRR-RLK subfamily of the RLK family. SMART (see Text Footnote 2) analysis showed that OsWSS1 contains an N-terminal signal peptide domain (SP), seven LRR domains, a transmembrane domain (TM), and a serine/threonine kinase domain (Figure 5A and Supplementary Figure 6).

A total of 27 OsWSS1 homologous proteins (more than 60% identity with OsWSS1) were identified from different plant species to determine their evolutionary relationship. Phylogenetic analysis showed that OsWSS1 homologs were conserved in both monocots and dicots, and *OsWSS1* was closely related to the homologous genes in *S. bicolor*, *Z. mays*, and *T. aestivum* (Figure 5C). In addition, multiple alignment analysis of protein sequences revealed that most OsWSS1-like proteins have a highly conserved serine/threonine kinase domain (Supplementary Figure 7). Multiple sequence alignment and motif analysis revealed that the Leu396Pro amino acid substitution site in the *oswss1* mutant was located in a highly conserved LRR region (Figure 5B), suggesting that this site is critical for the function of OsWSS1.

### *OsWSS1* Is Constitutively Expressed and the Encoded Protein Localizes to the Plasma Membrane

**FIGURE 6:**
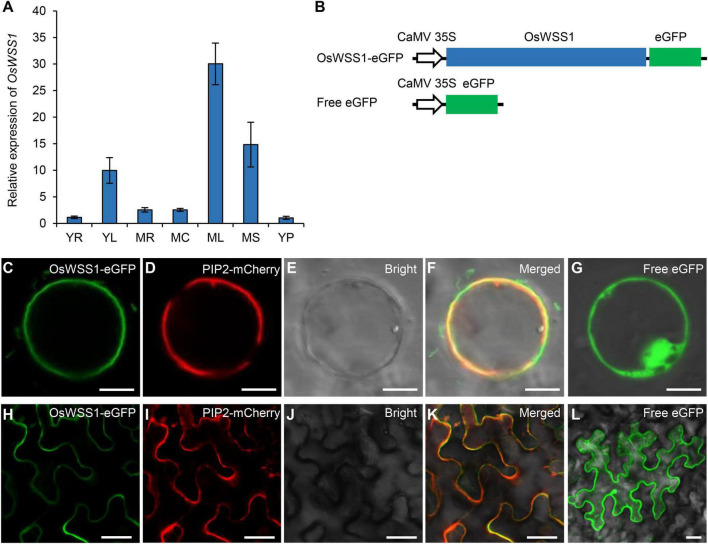
Expression pattern of *OsWSS1* and subcellular localization of the OsWSS1-eGFP protein. **(A)** Expression pattern of *OsWSS1* in different tissues of the wild type plants. YR, Young root; YL, young leaf; MR, mature root; MC, mature culm; ML, mature leaf; MS, mature sheath; YP, young panicle. *OsWSS1* expression was normalized using the *OsActin* gene as an internal control. *OsWSS1* expression of YR was normalized to 1 after the normalization with the value of *OsActin*. Values are mean ± SD (*n* = 3). **(B)** A schematic diagram of the constructs used for subcellular localization. **(C–F)** Subcellular localization of OsWSS1 in rice protoplast. The fusion protein (OsWSS1-eGFP) **(C)** was co-expressed with the plasma membrane marker (PIP2-mCherry) **(D)**. **(G)** Subcellular localization of the control (Free-eGFP) in rice protoplast. **(H–K)** Subcellular localization of OsWSS1 in epidermal cell of *Nicotiana benthamiana* leaves. The fusion protein (OsWSS1-eGFP) **(H)** was co-expressed with the plasma membrane marker (PIP2.1-mCherry) **(I)**. **(L)** Subcellular localization of the control (Free-eGFP) epidermal cell of *N. benthamiana* leaves. Scale bars: 5 μm **(C–G)**; 15 μm **(H–L)**.

The qRT-PCR was performed to detect the relative expression level of *OsWSS1* in different organs of the WT plants. *OsWSS1* was constitutively expressed in all tissues at young seedlings stage and heading stage, its expression level was relatively lower in roots, stems, and panicles, and relatively higher in leaves (Figure 6A). To further assess the transcript level of *OsWSS1* during leaf senescence, we tested *OsWSS1* expression in WT leaves at different developmental stages by qRT-PCR. The *OsWSS1* transcript levels were not significantly different in various ages and regions of WT leaves (Supplementary Figure 8), indicating that *OsWSS1* is not induced by senescence.

Using the Cell-PLoc 2.0 program^6^, OsWSS1 was predicted to be localized in plasma membrane. To confirm OsWSS1 localization, the expression vector of the *OsWSS1* and *eGFP* fusion was constructed and co-expressed with the plasma membrane marker PIP2-mCherry (Heng et al., 2018) in rice leaf sheath protoplasts (Figure 6B). Confocal laser-scanning microscopy revealed that the green fluorescent signal of OsWSS1-eGFP was indeed colocalized with PIP2 on the plasma membrane, while the negative control, 35S-eGFP, was localized at both the cytoplasm and nucleus (Figures 6C–G). Similar results were obtained in *Nicotiana benthamiana* leaf epidermal cells (Figures 6H–L). These results demonstrated that OsWSS1 is a plasma membrane protein.

### Mutation of *OsWSS1* Causes Early Leaf Senescence and Premature Cell Death

**FIGURE 7:**
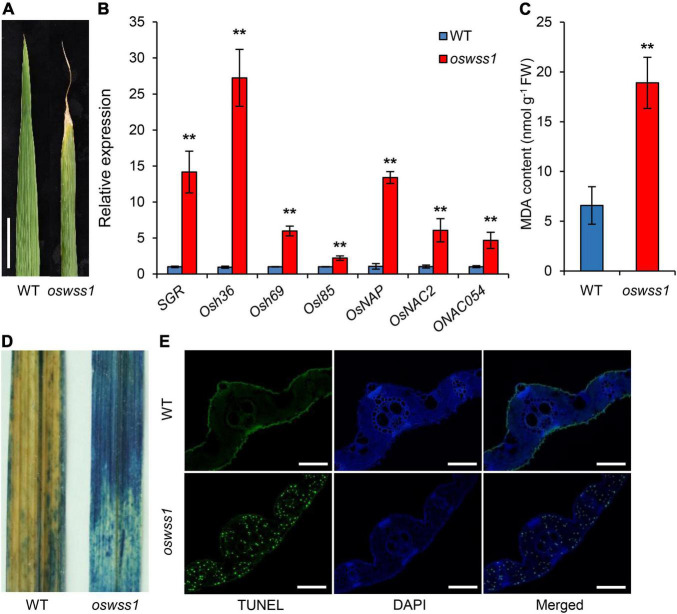
Mutation of *OsWSS1* causes early leaf senescence and premature cell death. **(A)** Leaf morphology between wild type (WT) and *oswss1* plants at the heading stage (118 DAS). Scale bar = 4 cm. **(B)** Transcriptional level analysis of senescence-related genes between WT and *oswss1* plants at the tillering stage (87 DAS) by qRT-PCR. *OsActin* was used as an internal control. The gene expression of WT was normalized to 1 after the normalization with the value of *OsActin*. Values are mean ± SD (*n* = 3). **P < 0.01 (Student’s *t*-test). **(C)** Comparison of the malondialdehyde (MDA) contents between WT and *oswss1* leaves at the tillering stage. Values are mean ± SD (*n* = 6). **P < 0.01 (Student’s *t*-test). **(D)** The trypan blue staining of leaves between WT and *oswss1* plants at the tillering stage. **(E)** TUNEL assay of WT and *oswss1* leaves at the tillering stage. Blue signal is 4′,6-diamino-phenylindole (DAPI) staining, green fluorescence represents TUNEL-positive signals. Scale bars = 50 μm.

Besides the water-soaked spots on leaves, *oswss1* mutant displays early leaf senescence (Figure 7A). The *oswss1* leaves exhibit water-soaked spots which subsequently developed into necrotic symptoms. Since the expression levels of many *SAGs* will change during leaf senescence, we determined the expression levels of *SAGs* in *oswss1* and WT plants by qRT-PCR. Results showed that the expression levels of all tested *SAGs* were significantly upregulated in *oswss1* (Figure 7B). In addition, the content of the lipid peroxidation product MDA was increased in *oswss1* by 1.4 times as compared with the WT (Figure 7C).

Trypan blue is an indicator of leaf cell death. After trypan blue staining, large area of the *oswss1* leaves was stained blue while only small area of the WT leaves was stained blue (Figure 7D). The TUNEL assay can detect DNA fragmentation that results from apoptotic signaling cascades by labeling the terminal end of nucleic acids. We accordingly used TUNEL assays to determine cell death in the *oswss1* mutant. As expected, there were few TUNEL positive signal spots in the WT leaves, whereas there were many strong and high TUNEL positive signals in *oswss1* leaves (Figure 7E and Supplementary Figure 9). These data indicated that DNA damage was more serious in *oswss1* leaves compared with that of WT leaves. Overall, these results suggested that the *oswss1* mutant stimulated premature cell death (PCD) and accelerated the leaf senescence.

### Reactive Oxygen Species Is Highly Accumulated in the *Oswss1* Leaves

**FIGURE 8:**
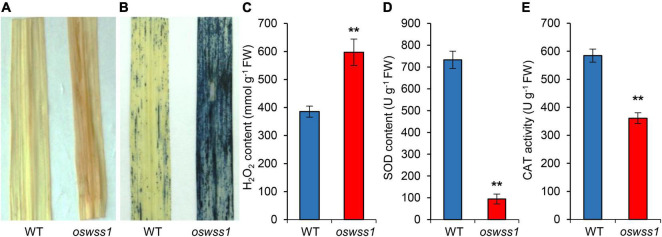
Reactive oxygen species is highly accumulated in the *oswss1* leaves at the tillering stage (87 DAS). **(A)** 3,3′-diaminobenzidine (DAB) staining of leaves between wild type (WT) and *oswss1* plants. **(B)** The nitroblue tetrazolium (NBT) staining of leaves between WT and *oswss1* plants. **(C–E)** Quantification of H_2_O_2_ contents **(C)**, SOD activity **(D)**, and CAT activity **(E)** in leaves between the WT and *oswss1* plants. Values are mean ± SD (*n* = 6). **P < 0.01 (Student’s *t*-test).

The ROS accumulation usually causes severe damage to most tissues and cells in plant leaves, leading to leaf senescence (Khanna-Chopra, 2012; Xu et al., 2022). To detect the ROS level in the water-soaked spots of the *oswss1* mutant, we used DAB and NBT staining to detect H_2_O_2_ and O_2_^–^ accumulation, respectively. The *oswss1* leaves showed dark brownish red color while the WT leaves exhibited white color after DAB staining (Figure 8A). NBT stained blue spots were densely distributed in the *oswss1* leaves and sparsely distributed in WT leaves (Figure 8B). We also measured the H_2_O_2_ concentration in the leaves and results showed the H_2_O_2_ content in *oswss1* leaves was increased by 40.4% compared with that of WT leaves at the tillering stage (Figure 8C). These data showed that ROS accumulated at a higher level in *oswss1* leaves compared with that of WT. To determine whether hyper-accumulation of ROS would activate the antioxidant enzymes, the activities of SOD and CAT were detected. The activities of SOD and CAT of *oswss1* leaves were significantly lower compared with that of the WT control (Figures 8D,E). Our findings suggested that mutation of *OsWSS1* results in ROS accumulation in rice leaves.

### The Expression of Genes Related to Chloroplast Development, Chlorophyll Synthesis and Photosynthesis Altered in *Oswss1* Mutant

**FIGURE 9:**
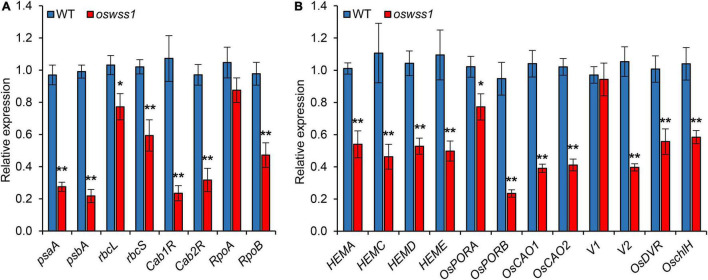
The expression of genes related to chloroplast development, chlorophyll synthesis and photosynthesis altered in *oswss1* mutant at the tillering stage (87 DAS) by qRT-PCR. **(A)** The expression of genes involved in photosynthesis between wild type (WT) and *oswss1* mutant. **(B)** Transcriptional level analysis of expression of genes involved in chlorophyll synthesis and chloroplast development between WT and *oswss1* mutant. *OsActin* was used as an internal control. The gene expression of WT was normalized to 1 after the normalization with the value of *OsActin*. Values are mean ± SD (*n* = 3). *P < 0.05, **P < 0.01 (Student’s *t*-test).

To investigate the role of OsWSS1 in chloroplast development, the expression of genes related to chloroplast development and photosynthesis (*psaA*, *psbA*, *rbcL*, *rbcS*, *Cab1R*, *Cab2R*, *RpoA*, and *RpoB*) (Huang et al., 2020) was compared between WT and *oswss1* leaves. The expression of all the tested genes except *RpoA* was significantly lower in *oswss1* as compared with that of the WT (Figure 9A). Since the chlorophyll content of *oswss1* decreased (Figure 1B), the expression of 12 chlorophyll biosynthesis-related genes (*HEMA*, *HEMC*, *HEMD*, *HEME*, *OsPORA*, *OsPORB*, *OsCAO1*, *OsCAO2*, *V1*, *V2*, *OsDVR*, and *OschlH*) were compared between WT and *oswss1* leaves. The expression of chloroplast biosynthesis-related genes, such as *OsPORB* (Sakuraba et al., 2013), *OsCAO1* (Lee S. et al., 2005), *OsCAO2* (Lee S. et al., 2005), and *V2* (Sugimoto et al., 2007), were significantly less in *oswss1* leaves than that of the WT (Figure 9B). These data suggest the *OsWSS1* mutation (Leu396Pro amino acid substitution) affects the expression of genes related to chloroplast development, photosynthesis, and chloroplast biosynthesis. These results also imply that OsWSS1 is involved in chloroplast development in rice.

## Discussion

### *OsWSS1* Encodes a Transmembrane Kinases Which Is Essential for Normal Growth and Development of Rice

Despite the extensive studies of some RLKs (including TMK) on regulation of plant growth and development (Clark et al., 1997; Diévart and Clark, 2004; Lee L. Y. et al., 2012; He et al., 2021), up to the present, the function of TMK controlling plant leaf senescence and cell death remains unknown. In this study, we have revealed that rice OsWSS1, a TMK protein, affects plant leaf senescence and cell death. The mutant *oswss1* showed reduced chlorophyll content, abnormal chloroplast development, reduced plant height, reduced seed-setting rate, and decreased 1,000-grain weight (Figures 1–3 and Supplementary Figure 1). Unlike rice, there is no significant difference of plant growth and development between *Arabidopsis TMK* T-DNA insertion single mutant and the wild type (Dai et al., 2013). This is possibly due to the difference of redundancy of TMK genes between *Arabidopsis* and rice. There are four *TMK* genes in *Arabidopsis* (Dai et al., 2013) but only one *TMK* gene in rice genome. The mutant phenotype of *oswss1* was complemented by expressing the wild type *OsWSS1* gene (Figure 4 and Supplementary Figure 4), demonstrating that OsWSS1 plays an important role in the growth and development of rice plants.

### *OsWSS1* Is Involved in the Leaf Epidermal Development and Chloroplast Development in Rice Leaves

The lesion-mimic and necrotic lesion in the leaf suggested that major changes in cell structure have occurred (Qiu et al., 2019; Cui et al., 2021). In this study, we observed changes in leaf cell structure between *oswss1* and WT plants. Paraffin section showed atrophy in the BCs and MCs in the water-soaked zone of *oswss1* leaves compared to the WT leaves (Figure 2B). Light microscope revealed the abnormal leaf epidermal cell shape in *oswss1* and the significantly changed cell structure (Supplementary Figure 3). It has been reported that the defective leaf epidermis is the main contributors to leaf rolling (Li et al., 2017). Thus, the rolled *oswss1* leaves may be caused by the defective leaf cell development in the epidermis. Moreover, the surface of water-soaked spots was severely shrunk in *oswss1* leaves (Figures 2E–G). The shrunken BCs (Figure 2B) explained the wrinkled leaf epidermal cell shape (Figure 2G and Supplementary Figure 3) observed on the *oswss1* mutant. Stomata is a key channel for water and gas exchange, which acts an important role to regulate water evapotranspiration and water utilization (Buckley, 2005). The *es1* (*early senescence1*) (Rao et al., 2015) and *ospls1* (*Oryza sativa premature leaf senescence 1*) (Yang X. et al., 2016) mutants exhibited excessive water loss due to increased stomatal density. However, the *oswss1* mutant exhibited lower stomatal density, altered stomatal morphology, and decreased cuticular papillae (Figures 2E–H), indicating that the water evapotranspiration capacity was reduced in *oswss1*. We speculate that the weakening of water transpiration leads to transpiration force reduction and organic transport obstruction, then would obstruct the leaf normal physiological activities, resulting in water-soaked spots of leaves.

Another typical feature of lesion formation is the change of chloroplast development and the chloroplast degradation (Cui et al., 2021). The chlorophyll content in the water-soaked spots of *oswss1* decreased by 30% compared to the WT (Figure 1B). In general, changes in chlorophyll content are associated with chloroplast development in rice (Zhang et al., 2017; Cui et al., 2019; Sun et al., 2019; He et al., 2020). As expected, TEM observation showed that chloroplast development was impaired, chloroplast number was reduced, and there were more degraded chloroplasts in the water-soaked zone of *oswss1* compared with that of the WT and the green part of *oswss1* plants (Figure 3). The formation of a functional chloroplast requires the coordinated expression of nuclear and chloroplastic genes (He et al., 2018; Chen et al., 2019). This process involves two types of RNA polymerases, nucleus-encoded polymerase (NEP) and plastid-encoded polymerase (PEP) (Jarvis and López-Juez, 2013). PEP transcribes chloroplast genes involved in photosynthesis, such as *psaA*, *psbA*, and *rbcL*; NEP largely transcribes housekeeping genes, such as *rpoA* and *rpoB*, which regulate plastid development at the early stages of plant growth (Shiina et al., 2005). In this study, we found the transcripts of PEP-dependent genes were significantly decreased, and the expression of chlorophyll synthesis-associated genes, including *HEMA*, *HEMC*, *HEMD*, *HEME*, *OsPORA*, *OsPORB*, *OsCAO1*, *OsCAO2*, *V2*, *OsDVR*, and *OschlH*, were also downregulated in the *oswss1* mutant (Figure 9). Overall, these results indicate that OsWSS1 may play a vital role in regulating leaf epidermal development and chloroplast development.

### Mutation of *OsWSS1* Promotes Reactive Oxygen Species Hyper-Accumulation and Cell Death

Reactive oxygen species are important signaling molecules and able to interact with many kinds of target molecules and metabolites, like DNA, proteins, lipids, and other cellular molecules, ROS accumulation can highly oxidative damage in cell structure and cellular components (Triantaphylidès et al., 2008; Ge et al., 2015). The *oswss1* exhibited hyper-accumulation of ROS in the water-soaked spots manifested by NBT and DAB staining, and measurement of H_2_O_2_ concentration (Figures 8A–C). ROS is generally in a dynamic balance in plant cells, and its content is determined by the delicate balance between ROS scavenging and ROS generation (Hu et al., 2011). SOD is the first defense line of plant ROS scavenging system to remove O_2_^–^ in the cell. CAT is another main ROS scavenging enzyme which catalyzes H_2_O_2_ to form H_2_O and O_2_ (Scandalios, 2002; Mittler et al., 2004). The activities of SOD and CAT were decreased in *oswss1* leaves (Figures 8D,E), suggesting that the hyper-accumulation of ROS may be caused by the reduction in the activity of these two key enzymes, thereby resulting in the water-soaked spots phenotype of *oswss1*. Taken together, these findings suggest that OsWSS1 plays a key role in maintaining ROS homeostasis during rice leaf development and chloroplast development. In addition, ROS can act as a signal to activate defense and offer wide possibilities for broad-spectrum disease resistance (Qi et al., 2017), future studies can examine whether OsWSS1 is involved in the disease resistance in rice.

Reactive oxygen species are also important factors in triggering early leaf senescence and PCD in plants (Loor et al., 2010; Wang et al., 2013; Mittler, 2017; Chen et al., 2021). The *oswss1* mutant exhibited remarkable increased amounts of oxidative-stress-induced toxic compound MDA, enhanced PCD (Figures 7C–E and Supplementary Figure 9). During leaf senescence, the expression of some *SAGs* is promoted (Lim et al., 2007; Yang et al., 2020). The results also showed that the expression levels of *SAGs*, *SGR* (Jiang et al., 2011), *Osh36*, *Osh69*, *Osl85* (Lee et al., 2001), *OsNAP* (Liang et al., 2014), *OsNAC2* (Mao et al., 2017), and *ONAC054* (Sakuraba et al., 2020) were significantly upregulated in the *oswss1* mutant (Figure 7B). Collectively, we predicted that the early leaf senescence of *oswss1* was caused by ROS-mediated PCD. In addition, leaf senescence is also influenced by various phytohormones, including abscisic acid (ABA) and jasmonic acid (JA) (Lim et al., 2007; Liang et al., 2014). The *Arabidopsis* TMK1 and TMK4 act as key regulators in the signaling cascades of ABA (Li et al., 2021; Yang et al., 2021); the *Nicotiana tabacum NtTMK1* was strongly induced by the treatment with JA (Cho and Pai, 2000), thus it is possible that *OsWSS1* is involved in the regulation of leaf senescence partially by modulating the signaling responses of phytohormones.

### The Putative Molecular Mechanism of OsWSS1 in Regulation of Reactive Oxygen Species Homeostasis

Plant LRR-RLKs are important membrane receptors that regulate plant development by sensing various ligands (Man et al., 2020). The LRR domain binds to ligands and transmits extracellular signals to the downstream (Chen, 2021). There was a point mutation in the 7th LRR domain of OsWSS1, in which the hydrophobic residue Leu396 was replaced by Pro amino acid in *oswss1* mutant (Figure 5B and Supplementary Figure 7). The hydrophobic residues play key roles in protein folding, ligand-protein and protein–protein binding, protein-nucleic acid interactions (Southall et al., 2002; Matsushima et al., 2005). Several pieces of evidence suggest that LRR-RLKs play a role in ROS homeostasis (Ouyang et al., 2010; Lin et al., 2020). Overexpression of *OsSIK2* in rice reduced ROS accumulation by enhancing the activities of POD, SOD, and CAT (Ouyang et al., 2010). *OsSTLK*-overexpressing rice plants remarkably reduced ROS concentration by upregulating ROS-scavenging activities (Lin et al., 2020). In this study, the *oswss1* mutant exhibits hyper-accumulation of ROS and lower SOD and CAT activities (Figures 8C–E). The expression levels of *OsWSS1* were not significantly different between WT and *oswss1* mutant (Supplementary Figure 5F), and there was no distinct difference in *OsWSS1* expression during leaf senescence (Supplementary Figure 8), suggesting that the expression level of *OsWSS1* may not affect the aging process. Based on these results, we speculate that OsWSS1 probably stimulates the activities of the SOD or CAT and regulates ROS homeostasis through phosphorylation and activation of unknown proteins.

## Data Availability Statement

The original contributions presented in the study are included in the article/Supplementary Material, further inquiries can be directed to the corresponding authors.

## Author Contributions

KZ, JX, CW, and ZJ conceived and designed the research. JX, FX, FW, and YZ performed the experiments. TZ, ZJ, YT, and ZW participated in data analysis. JX, KZ, and TZ wrote the manuscript. All authors contributed to the article and approved the submitted version.

## Conflict of Interest

The authors declare that the research was conducted in the absence of any commercial or financial relationships that could be construed as a potential conflict of interest.

## Publisher’s Note

All claims expressed in this article are solely those of the authors and do not necessarily represent those of their affiliated organizations, or those of the publisher, the editors and the reviewers. Any product that may be evaluated in this article, or claim that may be made by its manufacturer, is not guaranteed or endorsed by the publisher.

## Footnotes

1
http://www.cbs.dtu.dk/services/TMHMM

2
http://smart.embl-heidelberg.de/

3
http://www.ebi.ac.uk/Tools/sss/ncbiblast/

4
https://rapdb.dna.affrc.go.jp/#

5
https://www.ncbi.nlm.nih.gov/

6
http://www.csbio.sjtu.edu.cn/bioinf/Cell-PLoc-2/
