# Supplementary material for: WATER-SOAKED SPOT1 Controls Chloroplast Development and Leaf Senescence via Regulating Reactive Oxygen Species Homeostasis in Rice
Source: Front Plant Sci. 2022 May 26;13:918673. doi: 10.3389/fpls.2022.918673 (PMC9178249; doi:10.3389/fpls.2022.918673)
Supplement: Supplementary file 1 [file Data_Sheet_1.docx]

Supplementary Materials

**WATER-SOAKED SPOT1 Controls Chloroplast Development and Leaf Senescence via Regulating ROS Homeostasis in Rice**

**Jiangmin Xu^1,2†^, Zhiyuan Ji^1†^, Chunlian Wang^1†^, Feifei Xu^1^, Fujun Wang^1,3^, Yuhan Zheng^1^, Yongchao Tang^1^, Zheng Wei^1^, Tianyong Zhao^2*^, Kaijun Zhao^1*^**

^1^National Key Facility for Crop Gene Resources and Genetic Improvement, Institute of Crop Sciences, Chinese Academy of Agricultural Sciences, Beijing 100081, China

^2^State Key Laboratory of Crop Stress Biology for Arid Areas, College of Life Sciences, Northwest A&F University, Yangling, Shaanxi 712100, China

^3^Institute of Rice Research, Guangdong Academy of Agricultural Sciences, Guangzhou 510640, China

*** Correspondence:**Kaijun Zhao, zhaokaijun@caas.cn; Tianyong Zhao, [tzhao2@nwafu.edu.cn](mailto:tzhao2@nwafu.edu.cn)

**^†^**The authors contribute equally to this work.

# Supplementary Figures


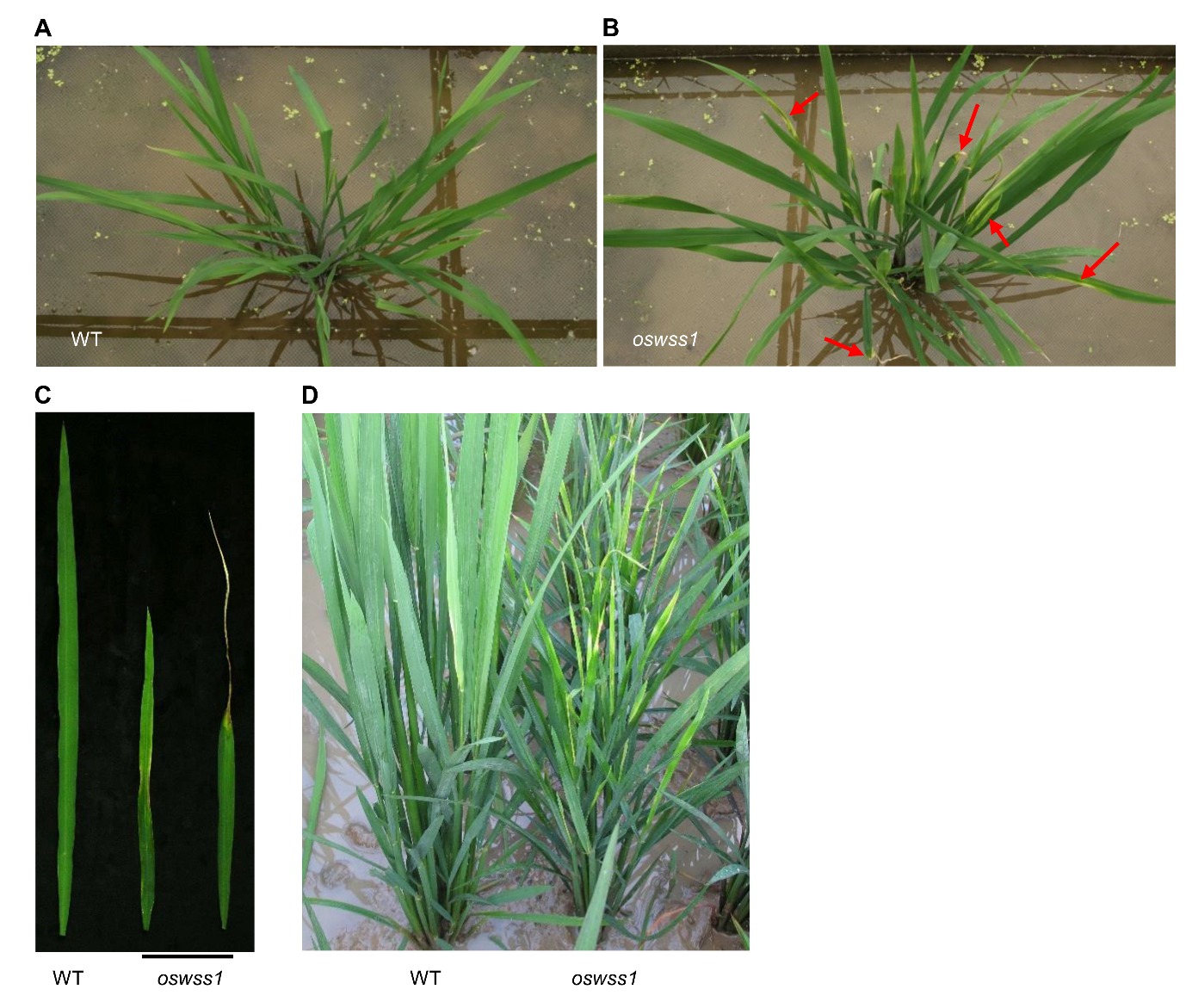


**Supplementary Figure 1.** Comparison of gross morphology between wild type (WT) and *oswss1* plants in the paddy field. **(A)** Phenotype of WT at the initial tillering stage (60 DAS). **(B)** Phenotype of *oswss1* at the tillering stage. Arrows indicate the water-soaked spots on leaves. **(C)** Leaf phenotype of WT and *oswss1* plants at the initial tillering stage. **(D)** Phenotype of WT and *oswss1* plants at the booting stage (105 DAS).


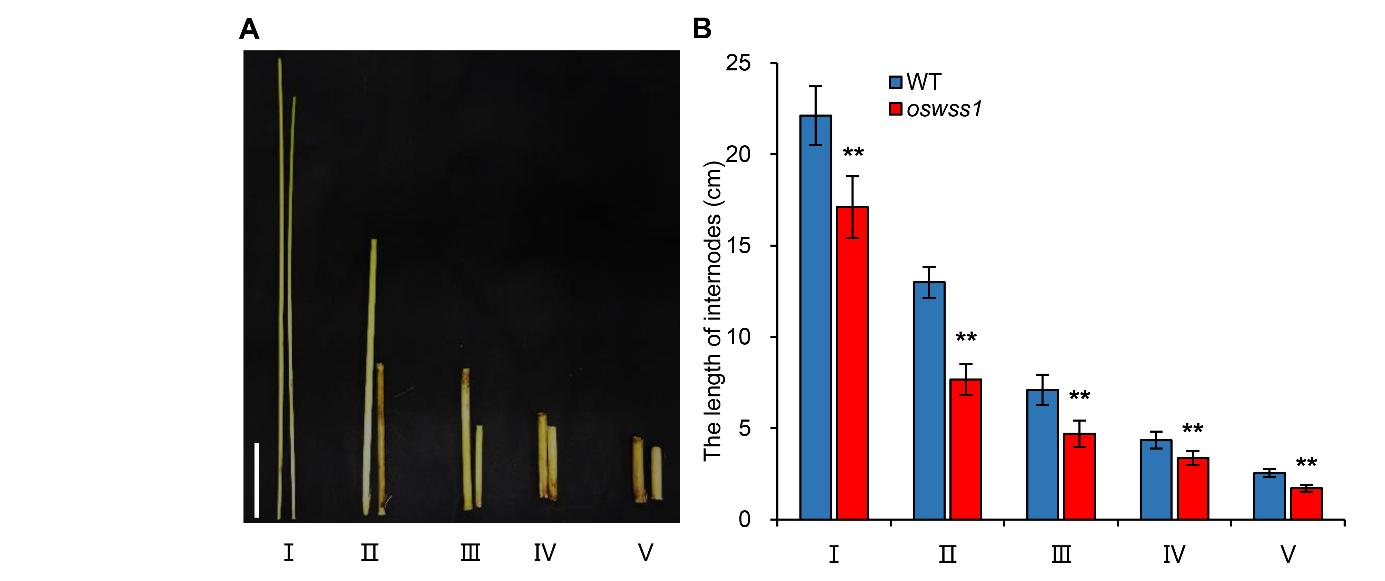


**Supplementary Figure 2.** Comparison of internode length between wild type (WT) and *oswss1*. **(A)** Comparison of main stems between WT (left) and *oswss1* (right). Scale bar = 4 cm. **(B)** Comparison of internode lengths of main stems between WT and *oswss1* at the mature stage (150 DAS). Values are mean ± SD (*n* = 6). ***P* < 0.01 (Student’s *t*-test).


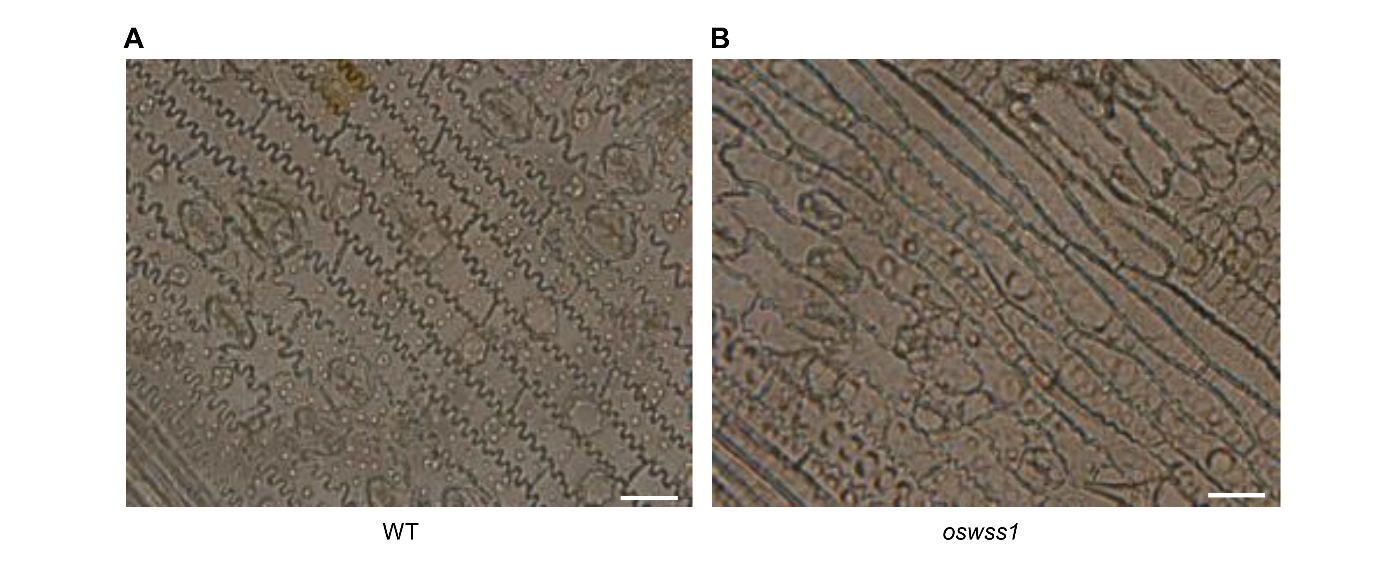


**Supplementary Figure 3.** Comparison of the leaf epidermal cell between the wild type (WT) **(A)** and *oswss1* **(B)** at the tillering stage (87 DAS). Scale bars = 5 µm.


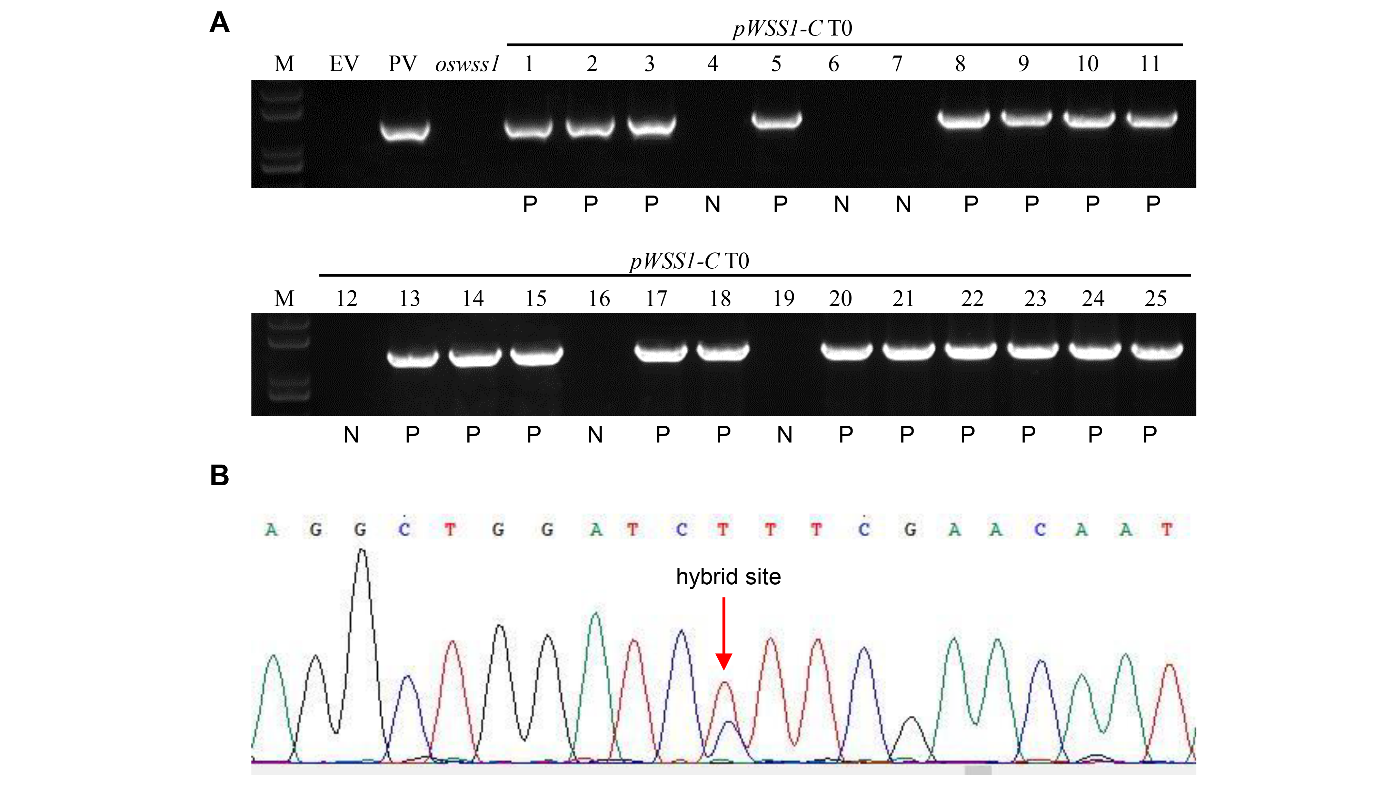


**Supplementary Figure 4.** Molecular detection of genetic complementary transgenic rice lines in the T0 generation. **(A)** Genomic DNA PCR analysis of complementary transgenic lines with primers WSS1-6F/1300-R. M: marker; EV: empty vector; PV: positive vector; P: positive plant; N: negative plant. **(B)** Heterozygote sequence of *pWSS1-C1* complementary transgenic plant.


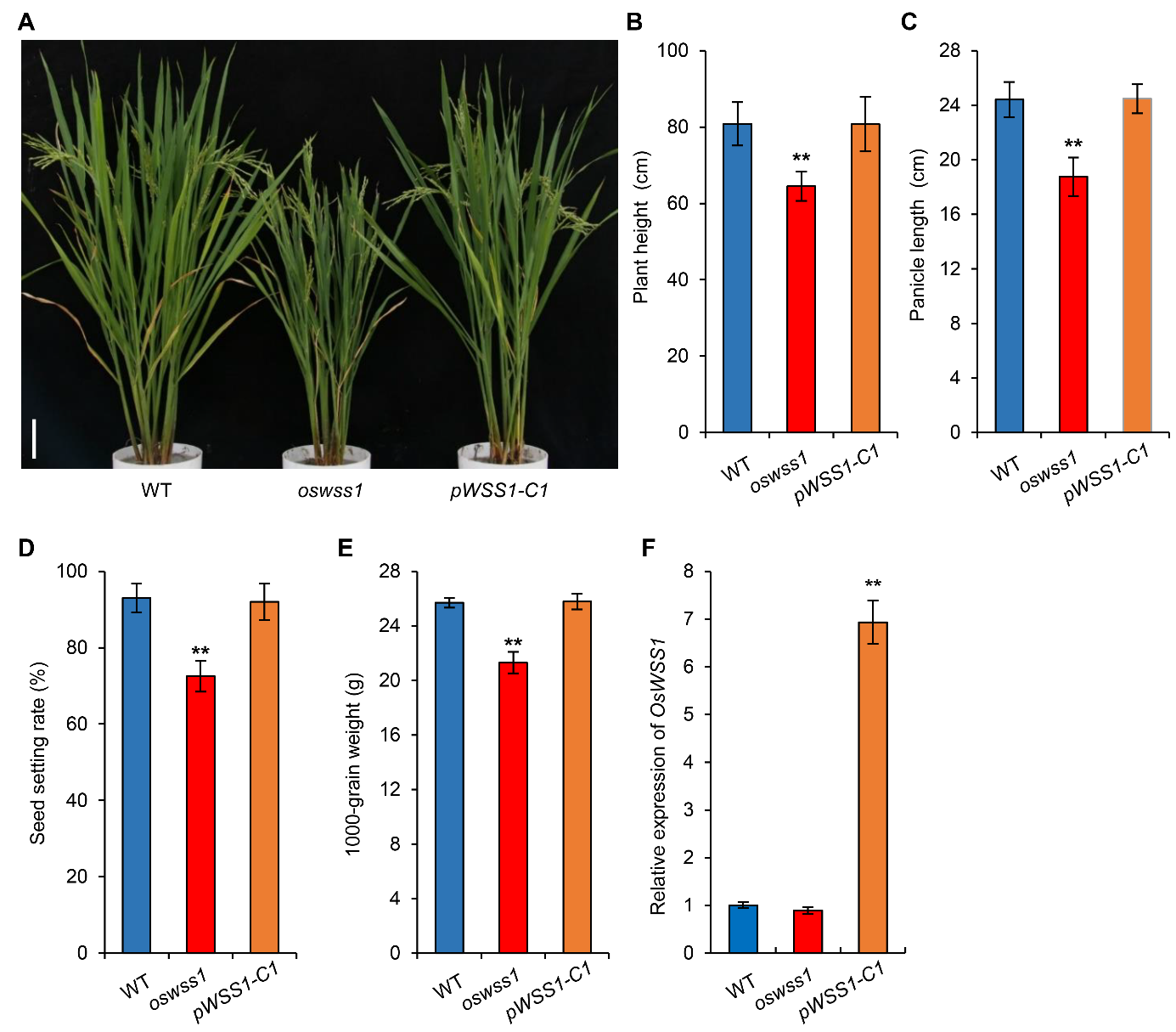


**Supplementary Figure 5.** Phenotype and major agronomic traits of the *OsWSS1* complementation plant *pWSS1-C1*. **(A)** Comparison of phenotype among wild type (WT), *oswss1* and complementation plant *pWSS1-C1* at the heading stage (118 DAS). Scale bar = 8 cm. **(B-E)** Major agronomic traits of WT, *oswss1* and *pWSS1-C1*. Values are mean ± SD (*n* = 10). **(F)** The expression level of *OsWSS1* in WT, oswss1 and *pWSS1-C1*. *OsActin* was used as an internal control. The *OsWSS1* expression of WT was normalized to 1 after the normalization with the value of *OsActin*. Values are mean ± SD (*n* = 3). ***P* < 0.01 (Student’s *t*-test).


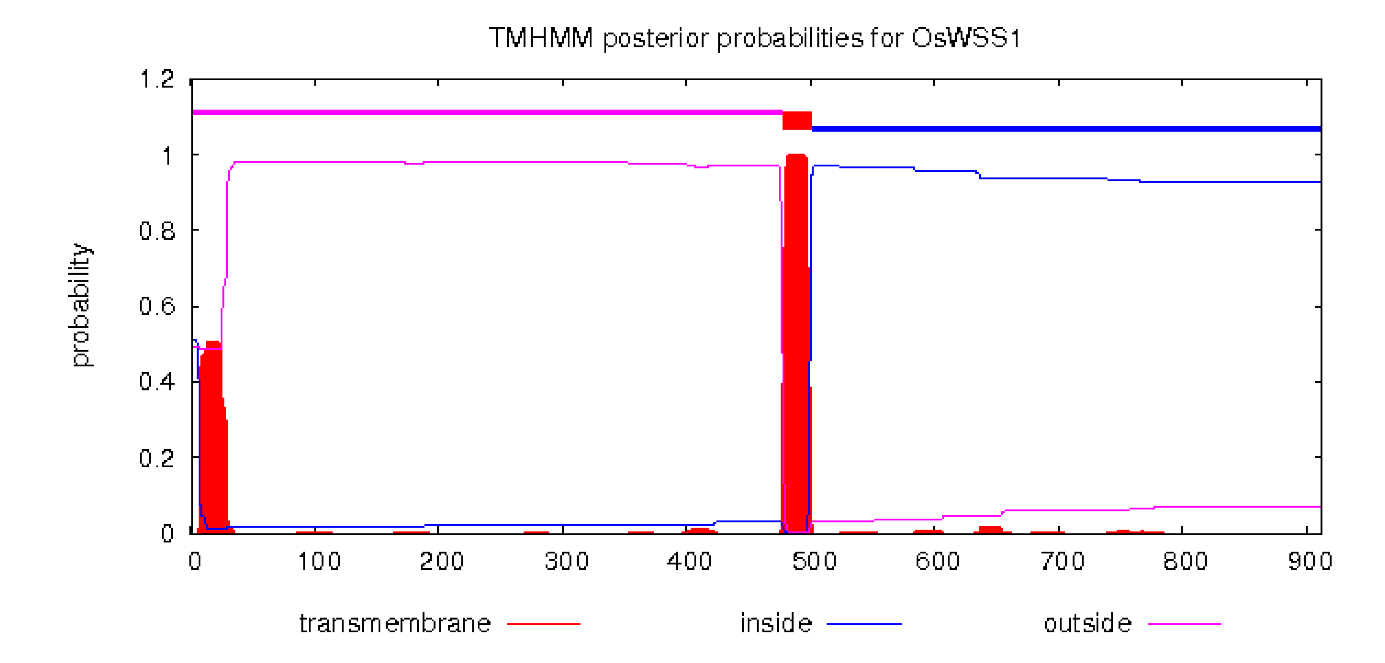


**Supplementary Figure 6.** TMHMM posterior probabilities for OsWSS1. The red area represents the transmembrane region.


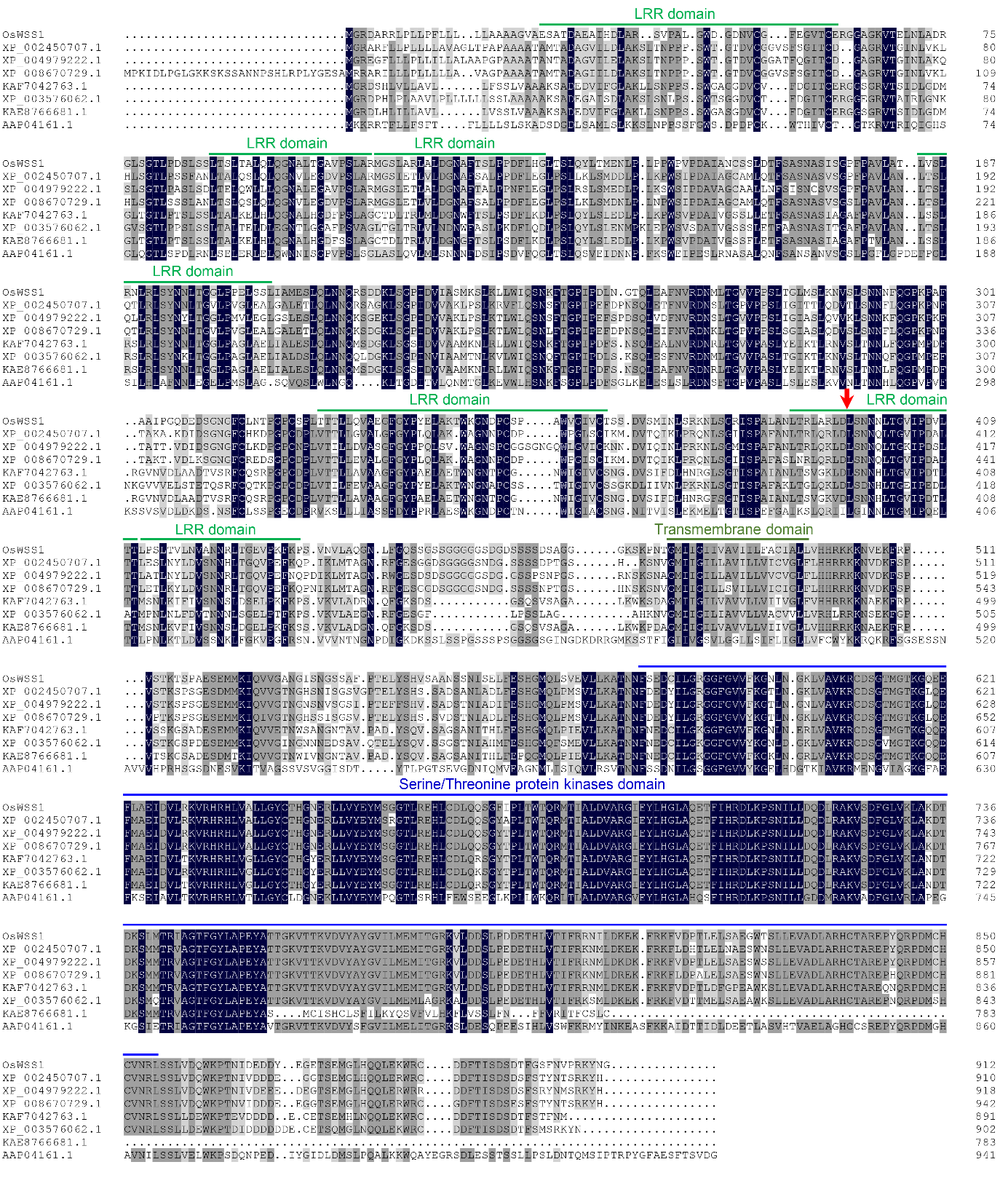


**Supplementary Figure 7.** Protein sequence alignment of OsWSS1 and its homologs from several species. Protein sequence alignment was performed among *OsWSS1* and its homologs from *Sorghum bicolor* (XP_002450707.1), *Setaria italic* (XP_004979222.1), *Zea mays* (XP_008670729.1), *Triticum aestivum* (KAF7042763.1), *Brachypodium distachyon* (XP_003576062.1), *Hordeum vulgare* (KAE8766681.1) and *Arabidopsis thaliana* (AAP04161.1). Arrow indicates the position of the amino acid transition.


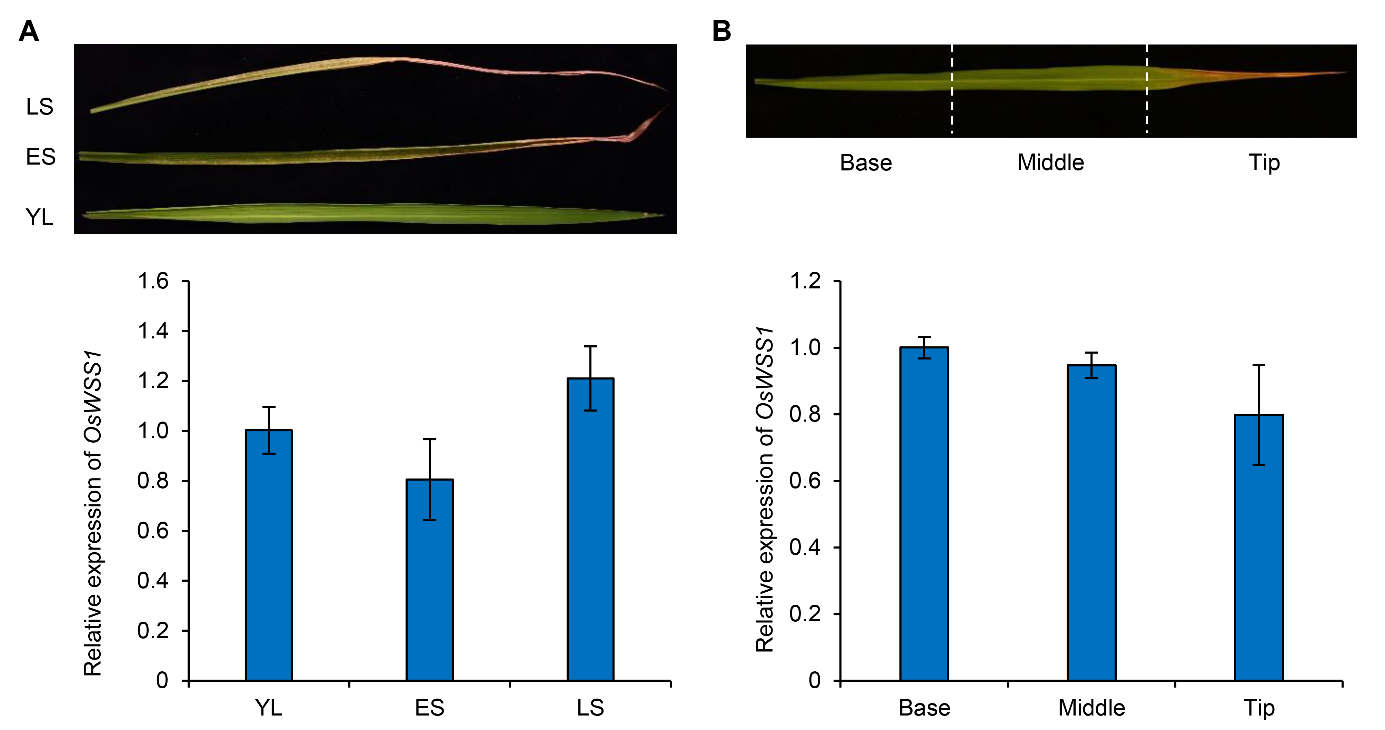


**Supplementary Figure 8**. Expression patterns of *OsWSS1* during leaf senescence determined by qRT-PCR. **(A)** The relative transcript levels of *OsWSS1* in various ages of wild type (WT) leaves. **(B)** The relative transcript levels of *OsWSS1* in tip, middle and basal part of senescing WT leaves. *OsActin* was used as an internal control. The *OsWSS1* expression of YL or Base was normalized to 1 after the normalization with the value of *OsActin*. YL, ES and LS: young leaves, early-senescing and late-senescence, respectively. Values are mean ± SD (*n* = 3).


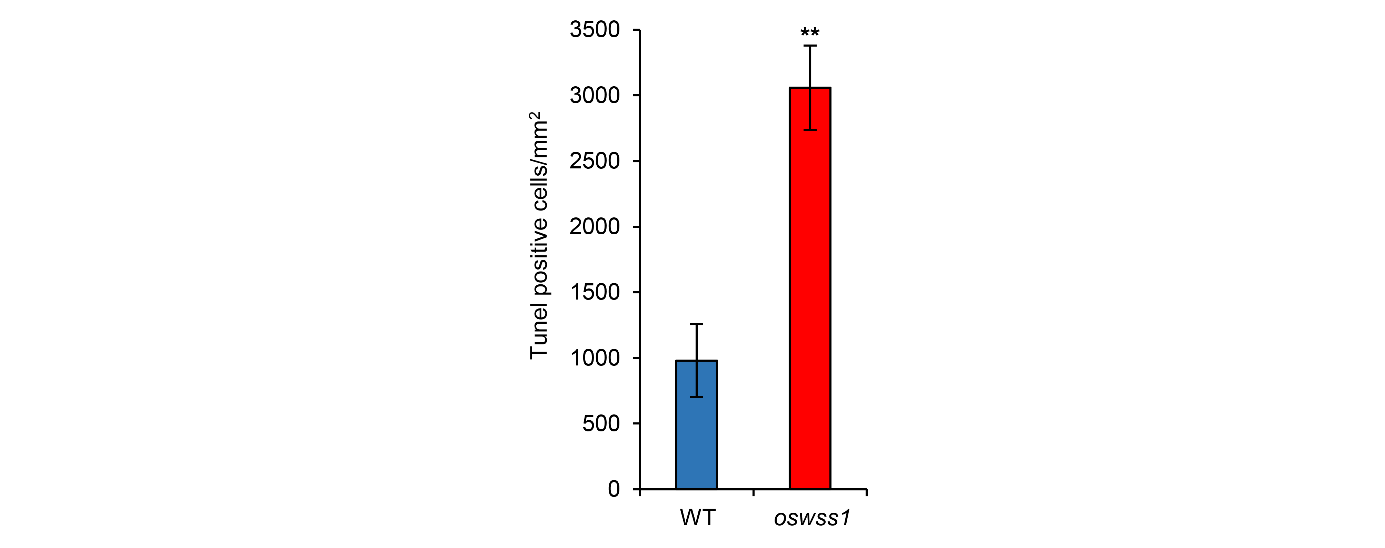


**Supplementary Figure 9.** Comparison of the numbers of TUNEL-positive cells in the wild type (WT) and *oswss1* mutant. Values are mean ± SD (*n* = 10). ***P* < 0.01 (Student’s *t*-test).

# Supplementary Tables

**Supplementary Table 1**. Cross populations for genetic analysis of the *oswss1* mutant.

| Cross | F_1_ | |  | F_2_ | | | | |  |
| --- | --- | --- | --- | --- | --- | --- | --- | --- | --- |
|  | Wild type | *oswss1* |  | Wild type | *oswss1* | Total | *P*-value | χ^2^(3:1) |  |
| JG30/*oswss1* | 10 | 0 |  | 169 | 45 | 214 | 0.1796 | 1.8006 |  |
| 02428/*oswss1* | 27 | 0 |  | 2553 | 787 | 3340 | 0.0551 | 3.6790 |  |
| NIP/*oswss1* | 17 | 0 |  | 1262 | 397 | 1659 | 0.3142 | 1.0128 |  |

**Supplementary Table 2**. Primers used for mapping and mutation analysis of *OsWSS1*.

| Name | Forward sequence (5’-3’) | Reverse sequence (5’-3’) |
| --- | --- | --- |
| Ch11-10 | ACAATCCGTACCTGCGGTG | GCTAGCTACTCCGTACCAAAA |
| Ch11-36 | AGATTGCTGGGTGTGTCAAA | CGCTATCGTCACGCCCTATT |
| Ch11-37 | TGGCGCTGTCATTGAAAACA | ATGCCATGTTCATGTCAGCA |
| Ch11-38 | CAAGCAGGTGGTGAAATGGG | AAGAACCAAGTGCTCAACCG |
| Ch11-40 | GGAAGAAAAGCCATTGACGA | GGCAGCTTCTAGGGTTAGGG |
| Ch11-42 | AGGCAACAACACCAACAATG | CATGACATGTGCAGCAGAAA |
| Ch11-44 | CATTACACAACCTGGAACTCG | TTGCTTGGCGCTGAGATATT |
| Ch11-123 | gggaaacggatcaagtctagc | caaacatagatgacggactgca |
| Ch11-141 | ggcggatatggatgtggata | gaattaggccattggatgga |
| ID11-12 | TGGTTGTTCCTACTGCCATCA | GGGGAAGTTGGAGATGACCAG |
| ID11-131 | TGGGACTGAGCTGAAAAGGA | TACGCTTTGCCCATTTTGCT |
| CAPS-WSS1 | CCAGAGGTCGGATGACAAGCTATCG | CTCTACCTAAGATGCAGTCCTCACTGAAG |

**Supplementary Table 3**. Primers used for vector construction and transgenic line test.

| Name | Primer sequence (5’-3’) | Restriction enzyme |
| --- | --- | --- |
| pWSS1-CF | gagctcggtacccggggatccAGGAAAAACCCCCTCTCTCTCAACTG | *BamH*I |
| pWSS1-CR | caggtcgactctagaggatccCGTGGATTGGTTGTTCGGTTTAAATGTGG | *BamH*I |
| WSS1-GFP-F | tacgaattcgagctcggtaccATGGGGAGGGACGCCCGC | *Kpn*I |
| WSS1-GFP-R | ctcgcccttgctcacggatccTCCATTGTACTTCCTTGGCACG | *BamH*I |
| WSS1-6F | GCCACTGTGTGAACCGACTA |  |
| 1300-R | GGTAACGCCAGGGTTTTCCCAGTCA |  |

**Supplementary Table 4**. Primer sequences used for quantitative real-time PCR.

| Name | Forward sequence (5’-3’) | Reverse sequence (5’-3’) |
| --- | --- | --- |
| OsActin | TGGCATCTCTCAGCACATTCC | TGCACAATGGATGGGTCAGA |
| OsWSS1 | AACCATACCAGAGGCCAGAC | GCTTGTCTCACCCTCGTAGT |
| SGR | AGGGGTGGTACAACAAGCTG | GCTCCTTGCGGAAGATGTAG |
| Osh36 | GCACGGAGGCGAACGA | TTGAGCGGTAGCACCCATT |
| Osh69 | CCACAACACGGATAACTT | GGTGAACACTATGGAACA |
| Osl85 | GAGCAACGGCGTGGAGA | GCGGCGGTAGAGGAGATG |
| OsNAP | AACCATTTCATCGCGAACAAC | CAGTGACGATCCCTGCAAGG |
| OsNAC2 | GAGAAGTCTGGCTGGGTCAT | AACACCCACTCGTTCTTGGA |
| ONAC054 | GGTTCGGGCAGATTTTCCGG | GAGAGCGAGGTGAAGGGATC |
| HEMA | CGCTATTTCTGATGCTATGGGT | TCTTGGGTGATGATTGTTTGG |
| HEMC | TGCTTGACTGCAAGTTCCCTTG | CTAGAGCCAACAATGTAGCATGG |
| HEMD | TGGAAGGCTGCTGGAAATCCTAAG | TCCTTGGAAGCTCTGAGGCCAA |
| HEME | AGGAGCAGGTGAGGGAGCT | TGGCGTCTGCAAGGTGAGAC |
| OsPORA | ATGGCTCTCCAAGTTCAG | TGGCTCACGCTAAGGAAC |
| OsPORB | CCGCAAGGAGGGAGCGGTG | CCCTCTTGGTGCTAAGGCCG |
| OsCAO1 | TTGGCTCAGTTAATGAGGGCAGAATCC | GGATGCGCACGTTGAGCATCTTTGTGG |
| OsCAO2 | GATCCATACCCGATCGACAT | CGAGAGACATCCGGTAGAGC |
| V1 | TGGAGGTCGGGACAGAGGA | CGAGGAGCACCACCATCAC |
| V2 | GCAGCAGATCCGTGATTACA | GCTGCTCCTTGAATGTCCAC |
| OsDVR | CAGGTCGAGACCGTCAAGAAC | ATGACCTGGATCGGCACCTTG |
| OsChlH | AACTGGATGAGCCAGAAGAGA | AAATGCAAAAGACTTGCGACT |
| psaA | GGAGGTGGCGAGTTAGTA | GATTTGCTTTATCGGGTAT |
| psbA | TATGGGTCGTGAGTGGGA | TTATGCTCTGCCTGGAAT |
| rbcL | CTTGGCAGCATTCCGAGTAA | ACAACGGGCTCGATGTGATA |
| rbcS | CAGCAATGGCGGCAGGAT | AGGGCACCCACTTGGAACG |
| Cab1R | AGATGGGTTTAGTGCGACGAG | TTTGGGATCGAGGGAGTATTT |
| Cab2R | TGTTCTCCATGTTCGGCTTCT | GCTACGGTCCCCACTTCACT |
| RpoA | CGCATCAATTTGCGTCAAAG | GTTAGCTATAGGTTGTGCCGTATCA |
| RpoB | CAAGTTTTCGGAGCCGAGAT | GCTAAAGATCCAGTAAGTCCAACG |

**Supplementary Table 5**. Major agronomic traits of the *OsWSS1*-complemented plants.

| Materials | Agronomic traits | | | |
| --- | --- | --- | --- | --- |
|  | Plant height (cm) | Panicle length (cm) | 1000-grain weight (g) | Seed-setting rate (%) |
| WT | 80.83±5.67 | 24.42±1.30 | 25.69±0.36 | 93.01±3.78 |
| *oswss1* | 64.50±3.83^**^ | 18.77±1.41^**^ | 21.30±0.78^**^ | 72.51±4.03^**^ |
| *pWSS1-C1* | 80.80±7.19 | 24.48±1.07 | 25.78±0.58 | 92.01±4.78 |
| *pWSS1-C2* | 79.80±6.94 | 24.58±1.37 | 25.27±0.81 | 91.45±4.99 |
| *pWSS1-C3* | 80.60±7.23 | 24.64±1.28 | 25.17±0.96 | 90.48±4.07 |
| *pWSS1-C5* | 80.20±5.37 | 24.68±1.40 | 25.07±0.60 | 90.01±3.46 |
| *pWSS1-C8* | 80.30±5.02 | 24.08±0.73 | 25.60±0.79 | 91.37±3.22 |
| *pWSS1-C9* | 81.00±4.06 | 24.22±0.93 | 24.93±0.34 | 93.15±3.99 |
| *pWSS1-C10* | 81.30±3.99 | 24.50±1.44 | 25.36±0.70 | 93.71±2.38 |
| *pWSS1-C11* | 81.80±5.81 | 24.66±0.53 | 25.24±0.60 | 93.50±2.62 |
| *pWSS1-C13* | 82.80±4.97 | 23.80±0.49 | 24.96±0.67 | 91.91±1.62 |
| *pWSS1-C15* | 81.70±5.02 | 23.98±0.70 | 25.36±0.68 | 91.92±2.28 |

The data in table are the average value ± standard deviation of 10 individual plants. ***P* < 0.01 (Student’s *t*-test).
